# An interview with Samuel Shem, author of ‘The House of God’

**DOI:** 10.1080/17571472.2017.1317424

**Published:** 2017-05-22

**Authors:** Claire Brash

**Affiliations:** Medical Student, Imperial College London, London, UK

## Abstract

Dr Stephen Bergman, Professor of medical humanities at New York University, writes under the name Samuel Shem. He is an acclaimed author of several novels, plays and textbooks, and his work has been translated into several languages –’The House of God’ his first novel has sold over 3 million copies. His work exposes the potential moral challenges of the medical workplace and the connection between values, good relationships and healing. In 2015 he visited the UK for medical humanities academic tour which included keynote talks at the RCGP 2015 Annual Conference and at the Royal Society of Medicine. Claire Brash interviewed him at the Royal Society of Medicine.

## Why this matters to me

A recent survey by the Student BMJ found that around 80% of medical students with mental health issues feel under-supported. And I wondered, since the topic of the RSM’s Medical Humanities Conference was physician resilience, what can you do early on in your career, to alleviate the stresses and strains of becoming a junior doctor?

## What led you to write the House of God?

Outrage, next question! I’d never written a novel before, but what I went through in the internship was so horrible, more horrible than the novel (some of the stuff I couldn’t put in there, you can imagine!), that after it was over, I started writing it for catharsis.

Six of the guys, who were mostly in the book, went on to their residency in the same place (I didn’t, I went on to psychiatry), so we would get together with a tape recorder, a couple of times, (one of those spool tape recorders) and just get drunk and talk.

So that led to my starting it, it started as a catharsis of the worst year of my life. I didn’t even think of getting it published and no publisher wanted it then finally somebody took it. Seven drafts later, it was a real book, you should have seen the first draft, unprintable, so angry.

## And do you feel that catharsis had the right effect for you?

Well, that’s a good question. I’m here, aren’t I? It kept me from suicide, that’s one good thing.

What happened was, (as I said, I’d never written a novel before). I had kind of an insight (well I’d written one comedy, a play, set in Oxford of all places, on Rhodes scholarship, but I’d never tried a novel) but the voice, (I hear voices all the time so …) came into my head and said ‘this has to ride on comedy, on humour, or no one is going to want to read it’ (because it’s so awful), and I mean, that’s the way we got through it, by the humour. What could you do? You were either going to laugh or you were going to bash your head in!

If you look at the book, the first third or more, 40% maybe is funny, really funny and really sexy. And then it gets darker. There are three times in their career that people read this book: one is (in the United States) before medical school, or in the first two years, and they think ‘oh this guy is so cynical, it’s not like this, what’s wrong with him? He’s a sicko!’ and then they walk onto the wards in year 3 and they say ‘oh my god, it’s all true’.

Then they read it as interns, or in clinic, they try to read it and they can’t get through it, cause when it gets to the dark part (people throwing themselves off buildings, stuff like that, going crazy, which happened), they put it down. I always have people say, ‘I tried to read it during my residency or internship and I couldn’t get through it, it was too awful’.

Then, years later, they sort of see it on their shelves sometimes and go back to it and say ‘oh my god’ and actually what also happens is (and people have been telling me this, on this tour), it wasn’t just a funny book, but there’s all this stuff in there that’s relevant, and it still is today.

I teach this at NYU, I teach a seminar on The House of God, absolutely it is relevant today as it was before.

## Do you feel that humour humanises medicine or do you think it could dehumanise the patients that it is directed at?

Well, it definitely humanises people who are doing the work, and the issue about humour in medicine (I get widely criticised for it, you know) ‘he’s making fun of patients’ or whatever, well that’s not true. You can’t let the patients know that you’re doing this.

There’s this great scene in the middle of the book where Berry comes in and the Fat Man and Roy are there and he’s showing her these four Roses, four women named Rose (who are in the Rose Room, it’s always occupied by four women named Rose). They’re GOMEREs named Rose, which was true. And Berry is absolutely astounded at the way he’s joking around and says ‘you can’t do that’ and the Fat Man steps in and says ‘you can’t let other people outside us know what we’re doing here’. The example he gave, (and I don’t know where it came from but it was right) is ‘do parents want to hear teachers criticising their children?’ – It’s the same thing. So I got blamed for something that was true, but we never transgressed anything.

## How much of the lexicon of the novel did you come up with yourself and how much was medical culture at the time?

Well I get blamed for all of it! The word GOMER (Get Out of My Emergency Room) was in usage, someone else used that where I trained in Boston, I did invent GOMERE though (a female GOMER), which I thought was rather clever. BUFF and TURF was in the lingo too, so I didn’t invent it, but I ran with it.

Most of the laws were ones which I invented or most of my guys throughout the year wrote down. But some of them only came to me when I was writing. Like one of the most important ones, ‘number 13: the delivery of medical care is to do as much nothing as possible’, which gets truer and truer every day actually. There’s even this massive study that came out of Sweden about meniscal knee surgery and it showed you’re just as well off not having the surgery, 5 years down the road. That’s a main source of income!

### How do you think culture has changed since you wrote the House of God – has it and is it for better or worse?

It’s changed for the better and it’s changed for the worse. (I can now talk about the NHS because I’ve talked about it a lot since being here, but I won’t). The US system of training has, believe it or not, got really better for trainees since the House of God.

There’s two things that really have a lot to do with this. I’ve been told this, and there have been seminars on this, not blowing my own horn but … Firstly, The House of God had a lot of impact on the doctors of my generation, who are now in charge, (of medical schools, of everything … they’re products the 60s like I was, so they’re not going to take the c**p without resisting). So there’s a volume that medical students should look at: ‘Return to the House of God: medical residency from 1978 TO 2008’ which is on my website (samuelshem.com). It’s a really good book, an anthology of all these things (if you really want to know about me, the last chapter ‘Living with Shem’ was by my wife, who was the model for Berry in the book, loosely!).

Secondly, the Libby Zion case, the Bell Report I think it’s called in the United States. Libby Zion was a young woman who came into an emergency room in the United States and was sort of misdiagnosed and died. The intern had been on for 30 h. Her father, who was a journalist, really got on it, and so, that led to a chain. It’s not clear that the intern did anything wrong but that led to a limitation of the hours that the interns can work (to 80) and they can’t work more than 16 h straight.

And that’s great for them, because they’ll have a life! We didn’t have a life, we were on call every other night for some of the year. Most of the year every third night, but some of the year, every other night! That meant you went in at 6 in the morning, you worked all the way round the clock until 6 at night, you went home and slept, and then you go in at 6 in the morning again. I mean it was insane, insane. Every third night is sort of tolerable, sort of, but every other night is not. So that’s a change that’s for the good for trainees.

There’s a question, a big debate, about whether it’s better for patient care. And I’ve flip-flopped on that a bit. Where it’s gotten worse, frankly, is the US health system, and I could go on and on, but I won’t. It’s a total mess, it’s a for-profit system, with a few little exceptions in certain states, (such as Massachusetts where I work), and Obamacare has made a little bit of ground, (but I wouldn’t exaggerate the coverage). The for-profit system is worse for doctors, (worse for interns, because they have to tick all of the boxes), worse for patients and only benefits the insurance companies.

‘I did a survey (I’m an NYU Professor in medical humanities and ethics) of what percentage of time interns (first year housemen), spend in front of screens, on their shift. Guess what the minimum percentage I came up with?’

## 30–40%?

80%! 80 was the minimum any interns (first year housemen) said! And they’re doing that under pressure from the system, they’d rather see patients. I could go on and on about it, but that’s a big deal.

It’s getting worse, and the last thing I’ll say (having said I wouldn’t talk about this too much) is that at that General Practice convention a couple of days ago, I’ve learned all about what’s going on.

## You are talking about junior doctor contracts!

You students have to get together and fight this, the NHS, the government is moving it towards the US service, for profit. And I said ‘you don’t want to go there, don’t go there!’ You have no idea how bad it is. You have to get it to the point that it’s clear, as a doctor, you can’t take the kind of care of patients that will result if this goes through. It’s not about money, it’s not about hours.

## One of the additional laws you added in was about empathy and learning empathy. is it possible to learn empathy?

That’s a very, very, very good question and well, I can give you a very short answer: yes, kind of. You learn empathy by it being shown to you by another doctor or intern. People are empathic, let’s go way back, they’ve often learned how to be in a relationship early on in life. They may have been doctors (people become doctors as they’ve often had diseases earlier in life and were treated by a doctor who was empathic to them and made them want to be in that connection) and the third thing is seeing doctors not acting empathically towards loved ones. Imagine sitting in a room with your aged mother (this happened to someone and they told me) and she’s in the bed and the team comes in and says ‘and how’s our gal today?’ asks all of these questions then they leave. I don’t act like that with my patients.

Secondly, and this is a longer answer, we had a very interesting session at Rhodes House in Oxford last week. And the first question that came up after I’d spoken for a while was ‘can you learn empathy?’ and I gave those kinds of answers. Then we were talking in the discussion afterwards, and finally, this rather young looking person said (there were three years of Rhodes scholars and it was the changeover, there were a couple who had just arrived) and this kid said ‘I’ve been very much on the achievement path all my life, and I’ve been focussed and I’m just wondering if being here in Oxford for just two or three years, if I should try new things? And so we talked a little bit more and I asked, ‘what year are you?’ and he said ‘I just arrived 3 years ago’. And everyone around him, (there were about 25 people), they all went ‘Ohhhh!’ – they felt so much for him, you know, that was a moment of empathy that no one will forget in that room.

You can forget knowledge, we all do (I forget everything) but you never forget what you understand, that’s the point. The real point about how you learn empathy is very simple, you learn it in a good relationship, it’s about not just learning empathy, but, if you’re lucky, learning mutual empathy. You know, you feel seen, the other person feels seen by you. And each person senses the other person feeling seen, and there’s a click.

My daughter Katie, when she first started having email, she had on her tag under her signature ‘it’s important to learn empathy, empathy is to walk a mile in another person’s shoes, that way you’re a mile away and you have their shoes’.

## A recent survey by the Student BMJ found that around 80% of medical students with mental health issues feel under supported. What can you do early on in your career, to alleviate the stresses and strains of going into a junior doctor career?

There’s one thing that you do, whether you have mental problems or not. (What are mental problems? They’re just another end of the curve. Unless you think all patients are rabid dogs. I mean, hallucinations aren’t that good, and Eat My Dust Eddie starts to hallucinate towards the end of the book, so we gave him a rest, which doubled our work). But there’s one thing, (and you should mention this): don’t isolate. The whole thing I wrote about, and everything I’m saying, is about the risk of isolation and the healing power of good connection. So don’t isolate!

Resilience comes from three things: community, a shared vision and good connections (in whatever relationships you’re in). So that’s a huge danger in medicine (men isolate more).

## Disclosure statement

No potential conflict of interest was reported by the author.

